# Comparing the impact of socio-demographic factors associated with traffic injury among older road users and the general population in Japan

**DOI:** 10.1186/1471-2458-12-887

**Published:** 2012-10-21

**Authors:** Takashi Nagata, Ayako Takamori, Hans-Yngve Berg, Marie Hasselberg

**Affiliations:** 1 Karolinska Institute, Department of Public Health Sciences, Division of Global Health, Stockholm, Sweden; 2 Kyushu University, Faculty of Medical Sciences, Department of Advanced Medical Initiatives, Fukuoka, Japan; 3 Kurume University Graduate School of Medicine, Kurume, Japan; 4 Swedish Transport Agency, Norrkoping, Sweden

## Abstract

**Background:**

The increasing number of older road users represents a public health issue because older individuals are more susceptible to traffic injury and mortality than the general population. This study investigated the association between socio-demographic factors and traffic injury and traffic mortality for the general population and among older road users in Japan.

**Methods:**

An ecological study was conducted using national data in Japan. Multivariate regression methods were applied to investigate the association of traffic injury and traffic mortality for the general population and among older road users with significant demographic, economic, road traffic, and medical/cultural factors.

**Results:**

Income per capita, total road length, and alcohol consumption per person were significantly associated with traffic injury and traffic mortality both for the general population and among older road users in Japan. Income per capita and alcohol consumption per person were negatively associated with traffic mortality for both groups. Meanwhile, for both groups, income per capita was positively associated with traffic injury, while total road length and alcohol consumption per person were negatively associated with traffic injury.

**Conclusions:**

The effects of socio-demographic factors on traffic injury and traffic mortality in Japan were similar for both the general population and older road users. The study results suggest that injury preventive measures designed for the general population will be beneficial also for older road users in Japan.

## Background

Traffic injury among older road users is becoming a major public health issue in industrialized countries [1-5]. This problem stems from varied phenomena: the number of older road users is increasing worldwide [6], and advances in transportation—including increased access to private vehicles—enable more frequent and easier travel for people 65 and older [7,8]. Additionally, the infrastructure and transportation systems of modern societies are mainly designed for a working population, and the needs of older road users are often not considered appropriately in their planning [7,8]. As a result, older road users may be more exposed to the risk of traffic injury [9].

Several studies have described significant associations between aging and traffic injury. Sensory, cognitive, and physical changes occur as part of the normal aging process and have been shown to increase the risk for traffic injuries [3,4]. Drivers age 80 and older have a 13-fold increase in mortality rate per kilometre driven due to fragility [10]. Fragility, as well as increased use of medication, among older road users complicates their trauma care and the clinical outcome of traffic injury. Indeed, compared with younger individuals, the skeletal structure of older road users is generally more vulnerable due to bone loss, and, as a result, less force can cause severe tissue damage and disruption, exaggerating the consequences of injury [11]. Further, increased use of medication might also increase the risk of road traffic crashes [12].

In the general population, factors related to the traffic environment, such as traffic density, number of vehicles, and types of vehicle, are associated with higher road traffic mortality [13,14]. Improving the infrastructure has proven to be a cost-effective measure that produces immediate reductions in crash and injury risk to older pedestrians and cyclists [2]. This finding indicates the need for a system designed to address the difficulties faced by older, more vulnerable, road users.

Socio-demographic factors are also known to contribute to traffic injury in the general population. Several studies have shown that low socioeconomic status is associated with traffic injury on an individual level [15], a regional level [16], and a national level [17]. In King County, Washington, United States (U.S.), the occurrence of pedestrian-motor vehicle collisions is associated with neighbourhood conditions and residential density [16]. A study from the Netherlands showed that higher income, greater traffic density, and increased availability of medical resources are significantly associated with lower traffic mortality in the total population [17]. Importantly, specific health conditions and social dependence among older road users may increase their risk for injury in the transport system compared to other age groups.

An additional risk factor for road traffic mortality in the general population is alcohol use [18]. Studies have shown that this risk applies to older road users as well [19,20]. An ecological study from Italy showed that the proportion of alcohol-impaired drivers in a population is associated with higher traffic mortality rates in the general population [13]. Alcohol is a risk factor of traffic injury both for total population and older road users [21].

Despite having the highest population aging rate in the world—more than 20% in 2010—no studies have examined the association between socio-demographic factors and traffic injury among older individuals in Japan. Interestingly, traffic injury in Japan is characterized by a low mortality rate but one of the highest morbidity rates among highly-industrialized countries. In 2008 the road traffic mortality rate was 4.7 per 100,000 people (4.3–14.4 per 100,000 among highly-industrialized countries) but the morbidity rate was as high as 744 per 100,000 people [22]. In contrast, the proportion of pedestrian mortalities is high compared to other countries. The annual number of pedestrian mortalities in Japan is more than 2500, or more than 30% of all traffic mortalities; in other high-income countries, such as the U.S., pedestrian mortalities account for about 13% of all traffic mortalities [23]. Half of all traffic mortality occurs among people age 65 and older, and the number of traffic injury is increasing for this age group [24]. This trend highlights the need for a better understanding of factors that are associated with the occurrence of traffic injury, and particularly whether these relationships differ for older road users compared to the general population.

This study investigated the association between socio-demographic factors and the occurrence of traffic injury in Japan. We hypothesized that the association between socio-demographic factors and traffic injury are different for the older population compared to the general population. In particular, we focused on socio-economic factors, the type of road traffic environment, and the level of alcohol consumption.

## Methods

This ecological, multi-group comparison, following a Morgenstern design, involved prefectures (n=47) as the units of analysis [25,26]. We tested the associations between traffic injury/mortality and demographic, economic, road traffic, and medical and cultural indicators. We used the most current information available from the following government agencies: the National Police Agency (NPA); the Bureau of Statistics; the Ministry of Land, Infrastructure, Transport and Tourism; the Ministry of Internal Affairs; the Fire and Disaster Management Agency; and the National Tax Agency. Selection of demographic, economic, road traffic, and medical and cultural indicators were guided by past studies and extensive reports on this topic. van Beeck et al. published one of the first articles using an ecological approach to study traffic injury [17]. The group used per capita income, degree of urbanization, unemployment rate, and the proportion of Roman Catholics in the population as socio-demographic factors. Additional factors they considered were traffic density, injury severity, the availability of highways and of cycling tracks, the mean length of ambulance transport, the regional availability of computerized tomography, and neurosurgery as regional characteristics. Later, La Torre et al. used the regional employment rate, the degree of regional urbanization, and the average per capita income by region as socio-demographic factors and prevalence of alcohol use (road behaviour), number of circulating vehicles (road vehicles), length of highways (road infrastructure), and availability of magnetic resonance imaging (trauma care) as other explanatory variables in their multiple linear regression models [13]. A similar study by Rivas-Ruiz et al. used percentage of high capacity roads, percentage of heavy vehicles, percentage of the population aged over 16 years that consume alcohol, mean annual precipitation, and index of the cultural and educational level [14].

Using the definitions of the World Health Organization and the Ministry of Health, Welfare and Labour, Japan, as guidelines, we defined people age 65 and over as “older road users” or “older people.”

Data on traffic injury and traffic mortality were obtained from the open domain of the National Police Agency, and were collected between January 1 and December 31, 2005. Japan is divided into 47 prefectures, or sub-national jurisdictions, from north to south; prefectures are governmental bodies larger than cities, towns, or villages. Japan’s data collection system for traffic injury/mortalities is managed by prefecture police headquarters before being sent to the NPA. In the NPA database, traffic mortality is defined as death within 24 hours after an incident, and traffic injury is defined as injury or death from an incident involving all types of vehicles. Traffic injury and mortality in the total population refer to those occurring among all people in Japan. Traffic injury and mortality among older populations are those occurring among people age 65 and older.

We compared traffic injury and mortality for the general population and among older road users with factors that were shown to be relevant in previous studies [13,14,17,27]. Prefecture-specific factors of interest were assessed in the following categories: (1) demographic factors, including general population, older road users (or population aged 65 years old and over), and proportion of older road users; (2) economic factors, including income per capita, unemployment rate, and degree of urbanization; (3) road traffic factors, including total road length, total number of registered vehicles, and total number of licensed drivers; and (4) medical and cultural factors, including number of physicians per 100,000 population, number of emergency patients transferred by ambulance per 100,000 population, alcohol consumption per person, and entrance rate of college/university after high school graduation. These 13 variables were considered as representative to reflect socio-demographic conditions in the prefectures.

### Statistical methods

First, we compared the prefecture-specific number of traffic injury/mortality for the general population and among older road users with the 13 independent factors described by bivariate Poisson’s regression analysis. Second, Pearson’s correlation analysis was performed to check the degree of mutual relationship among 13 independent variables and avoid assumption of multi-colinearity. Third, we employed multivariate regression methods, retaining significant demographic, economic, road traffic, and medical and cultural factors in the preceding section. Because traffic injury and mortality are relatively rare events, Poisson’s distribution was applied in the analysis [28,29]. We checked model fitting and adherence to regression modeling assumption manually. We also checked the interactions among explanatory variables retained at each step in the model-fitting procedure.

SAS 9.1 was used in the analysis, and p-values less than 0.05 were accepted as statistically significant. The study was approved by the Institutional Review Board at Kurume University, Japan.

## Results

**Figure 1:**
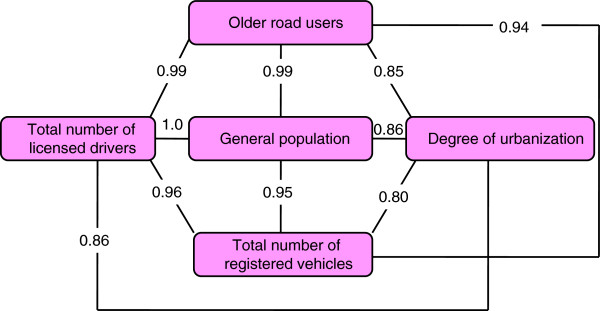
The associations of variables with high correlation of estimations

The number of traffic injuries and traffic mortalities for the general population and among older road users varied in prefectures (Table 1). The same patterns were observed for demographic, economic, road traffic, and medical and cultural factors (Table 1). Twelve of the thirteen explanatory factors (Table 2), except for total road length, displayed a significant initial bivariate association with traffic injury and traffic mortality both for the general population and for older road users. We found a positive association between the proportion of older road users in prefectures and the number of traffic mortalities for both the total population and older road users. We also identified a positive association between the degree of urbanization and the number of traffic injuries for both age groups. In contrast, a negative association was found between demographic, economic, road traffic, and medical/cultural factors and traffic injury and traffic mortality for both the total population and for older road users. Finally, we tested potential associations of several independent variables (total population, older road users, degree of urbanization, total number of registered vehicles, and total number of licensed drivers) with one another through high correlations of estimation (Figure 1).

**Table 1:** Traffic injury/mortality and demographic, economic, road traffic, and medical and cultural indicators in Japan

**Factor**	**Data Source**	**Median (Max - Min)**
Traffic mortality for total population	National Police Agency	118 (45 - 351)
Traffic injury for total population	National Police Agency	16863 (3502 - 91272)
Traffic mortality among older people	National Police Agency	56 (17 - 133)
Traffic injury among older people	National Police Agency	2179 (577 - 8564)
***Demographic***		
Total population (1,000 persons)	Bureaue of Statistics	1753 (607 - 12577)
Population among older people (1,000 persons)	Bureaue of Statistics	391 (146 - 2296)
Proportion of older people (%)	Bureaue of Statistics	22.5 (16.1 - 27.1)
***Economic***		
Income per capita (1,000 yen)	Bureaue of Statistics	2729 (2021 - 4778)
Unempoyment rate (%)	Bureaue of Statistics	4.0 (2.3 - 7.9)
Degree of urbanization	Ministry of Land, Infrastructure, Transport and Tourism	44.4 (24.2 - 98.0)
***Road traffic***		
Total length of road (KM)	Ministry of Land, Infrastructure, Transport and Tourism	253x106 (78x106 - 884x106)
Total number of registered vehicles	Ministry of Land, Infrastructure, Transport and Tourism	12.8x106 (4.5x106 - 48.6x106)
Total number of licensed drivers	National Police Agency	11.2x106 (3.8x106 - 70x106)
***Medical and cultural***		
Number of physicians per 100,000 population	Ministry of Health, Labor and Welfare	212 (134.2 - 282.4)
Number of emergency patients transferred by ambulance per 100,000 population	Ministry of Internal Affairs Fire and Disaster Management Agency	3503 (2702 - 5695)
Alcohol consumption per person (liters)	National Tax Agency	67.8 (54.0 - 99.5)
Entrance rate of college/university after high school graduation (%)	Bureaue of Statistics	46.5 (31.1 - 58.4)

**Table 2:** Bivariate assocation between traffic injury/mortality and demographic, economic, road traffic, and medical and cultural indicators in Japan

	**Traffic mortality for total population**			**Traffic injury for total population**			**Traffic mortality among older people**			**Traffic injury among older people**		
	**Coefficient**	**SE**	**P-value**	**Coefficient**	**SE**	**P-value**	**Coefficient**	**SE**	**P-value**	**Coefficient**	**SE**	**P-value**
***Demographic***												
Total population (1,000 persons)	-0.260	0.010	<.0001	-0.040	0.001	<.0001	-0.281	0.016	<.0001	-0.064	0.002	<.0001
Population among older people (1,000 persons)	-0.261	0.010	<.0001	-0.042	0.001	<.0001	-0.283	0.016	<.0001	-0.069	0.002	<.0001
Proportion of older people (%)	0.223	0.012	<.0001	-0.002	0.001	0.080	0.220	0.018	<.0001	0.017	0.003	<.0001
***Economic***												
Income per capita (1,000 yen)	-0.168	0.010	<.0001	-0.021	0.001	<.0001	-0.182	0.016	<.0001	-0.032	0.002	<.0001
Unempoyment rate (%)	-0.202	0.014	<.0001	-0.061	0.001	<.0001	-0.222	0.021	<.0001	-0.104	0.003	<.0001
Degree of urbanization	-0.158	0.012	<.0001	0.041	0.001	<.0001	-0.159	0.018	<.0001	0.034	0.003	<.0001
***Road traffic***												
Total length of road (KM)	0.019	0.011	0.083	-0.013	0.001	<.0001	0.008	0.016	0.612	-0.033	0.003	<.0001
Total number of registered vehicles	-0.237	0.010	<.0001	-0.023	0.001	<.0001	-0.250	0.016	<.0001	-0.048	0.002	<.0001
Total number of licensed drivers	-0.257	0.010	<.0001	-0.037	0.001	<.0001	-0.276	0.016	<.0001	-0.059	0.002	<.0001
***Medical and cultural***												
Number of physicians per 100,000 population	-0.086	0.011	<.0001	-0.007	0.001	<.0001	-0.102	0.017	<.0001	-0.029	0.003	<.0001
Number of emergency patients transferred by ambulance per 100,000 population	-0.265	0.011	<.0001	-0.041	0.001	<.0001	-0.295	0.017	<.0001	-0.064	0.002	<.0001
Alcohol consumption per person (liters)	-0.157	0.010	<.0001	-0.065	0.001	<.0001	-0.174	0.016	<.0001	-0.094	0.002	<.0001
Entrance rate of college/university after high school graduation (%)	-0.295	0.011	<.0001	-0.047	0.001	<.0001	-0.314	0.017	<.0001	-0.077	0.002	<.0001

After confirming the mutual relationship of the explanatory variables by using the estimation of Pearson’s correlation, we excluded the variables with high correlations, and then combined factors in stepwise multivariate regression modelling for predicting traffic injury and traffic mortality for the total population and older road users. Among the economic factors, income per capita remained significantly associated with both traffic injury and mortality, while unemployment rate and degree of urbanization were not significantly associated. When we introduced road traffic-related factors, only total road length was significantly associated with traffic injury, though this factor was not significantly associated with traffic mortality. We also conducted the analysis using other road traffic-related factors such as total highway length, total length of rural road, and total kilometres driven for motor vehicle travel in prefectures, and obtained similar results.

We then introduced medical and cultural factors; only alcohol consumption per person was significantly associated with traffic injury, though it was not significantly associated with road traffic mortality. Because of multi-colinearity, demographic factors were not selected in the model. The results of the multiple regression analysis were identical between the total population and older road users (Table 3). Income per capita and alcohol consumption per person were negatively associated with traffic mortality for both groups. In other words, income per capita and alcohol consumption per person might have a preventive effect on traffic mortality. Similarly, income per capita was positively associated with traffic injury, while total road length and alcohol consumption per person were negatively associated with traffic injury for both groups. In other words, income per capita might have an exposure effect on traffic injury, while total road length and alcohol consumption per person might have a preventive effect on traffic injury.

**Table 3:** Regression modeling: Predictors of traffic injury and traffic mortality in Japan

**Traffic mortality among older people**				**Traffic injury among older people**			
**Variable**	**Coefficient**	**SE**	**P-value**	**Variable**	**Coefficient**	**SE**	**P-value**
Income per capita	-0.144	0.02	<.0001	Income per capita	0.021	0.003	<.0001
Total road length	-0.003	0.016	0.832	Total road length	-0.048	0.003	<.0001
Alcohol consumption per person (liters)	-0.122	0.018	<.0001	Alcohol consumption per person (liters)	-0.110	0.003	<.0001
**Traffic mortality for the total population**				**Traffic injury for the total population**			
**Variable**	**Coefficient**	**SE**	**P-value**	**Variable**	**Coefficient**	**SE**	**P-value**
Income per capita	-0.131	0.012	<.0001	Income per capita	0.021	0.001	<.0001
Total road length	0.002	0.011	0.865	Total road length	-0.025	0.001	<.0001
Alcohol consumption per person (liters)	-0.101	0.012	<.0001	Alcohol consumption per person (liters)	-0.081	0.001	<.0001

## Discussion

This study found that income per capita, total road length, and alcohol consumption per capita were significantly associated with traffic injury and traffic mortality in Japan. Income per capita and alcohol consumption per person might have a preventive effect on traffic mortality for the general population and for older road users. However, income per capita might have an exposure effect on traffic injury, while total road length and alcohol consumption per person might have a preventive effect on traffic injury for both groups. Although we hypothesized that regional variations in socio-demographic factors would affect the occurrence or outcomes of traffic injury, we found little variation among prefectures.

The current study showed that economic factors had significant associations both with traffic injury and traffic mortality, but the associations were opposing: higher income was associated with more traffic injuries, but less traffic mortality. This was found for both the total population and older road users. Indeed, economic factors such as income per capita have been considered as determinants of disease morbidity and mortality [29]. Previous studies showed inverted U-shaped patterns between economic growth and traffic injury [30,31]. In most industrialized countries, traffic injuries have increased in accordance with economic growth, until reaching a plateau and declining [31-33]. As countries are industrialized and vehicle use increases, exposure to traffic-related risks and traffic injury also increases. Economic development, therefore, can both increase the risk for traffic injuries and have a preventive effect [34]. The preventive effect is connected with better-protected users, such as motor vehicles with stronger frames, airbags, seat belts, etc. This preventive effect may explain the negative association of income per capita on traffic mortality in this study.

Total road length, which was considered as one of the indicators reflecting the road infrastructure, was negatively associated with traffic injury but not associated with traffic mortality in this study. This finding suggests that road infrastructure might be preventive for traffic injury, but not for traffic mortality, in Japan because traffic injury and mortality tend to happen on different kinds of roads. Improvement of road infrastructure, such as better road design, more signals, etc., has played an important role in preventing traffic injury [35]. Indeed, Egan reported that the construction of new major urban roads between towns and out-of-town bypasses could reduce traffic injury, while other types of roads (e.g., access roads) had no impact on traffic safety [36]. Also, well-planned road infrastructures, including appropriate speed limits, roundabout traffic systems, and road median barriers, are preventive against traffic mortality [37-41]. However, maximum effort over the last two decades has consistently reduced traffic mortality, and, as a result, there may not be room for further improvement through additional road traffic infrastructure endorsement [42].

Interestingly, alcohol consumption was negatively associated with traffic injury and traffic mortality, which differs from previous studies [5,43]. Usually, higher alcohol consumption in a country correlates with more traffic injury and traffic morality. Evaluations of Japanese road safety and injury prevention programs initiated since the 1990s, including strict alcohol-impaired driving laws, a revised criminal law, and improvements to road infrastructures, vehicle designs, and the emergency medical system, indicate a positive result and show that the road traffic mortality rate has gradually declined [5,42-44]. Similar effects have been observed for the road traffic injury rate since 2005. In Japan, due to strict anti-alcohol driving policies and a well-developed public transportation system, less than 10% of road traffic mortality was associated with alcohol-impaired driving, and, as a consequence, alcohol may not be a strong contributor for traffic injury and traffic mortality in Japan [44]. The initiatives taken in Japan to reduce drunk-driving provide a model for other countries addressing the role of alcohol in road traffic injuries. On the other hand, alcohol consumption per capita in prefectures might not accurately represent alcohol intake in individuals; this phenomenon can be associated with ecological fallacy [45,46], described later in this article.

The results also indicated that associations between the studied socio-demographic factors and road traffic injuries were similar for the general population and older road users. Income per capita, total road length, and alcohol consumption per capita may have produced similar effects for both the total population and older road users because these factors influence both groups in the same way. Alcohol consumption occurs in people age 20 and older in Japan, including older road users.

To confirm the validity of the selected variables, we included several socio-demographic factors that were specific to older road users (or population age 65 years and over) such as medical cost per older person in prefectures (not shown in the article), number of older road users, proportion of older people, etc. However, these factors were not included in the final multivariate regression analysis due to their high correlation with selected factors.

Although our study had no way to control for regional differences in traffic law enforcement by the police, since the traffic safety policy and law enforcement in prefectures are centrally managed by the National Police Agency, variations between prefectures are expected to be quite low in Japan [24].

This study contains several limitations. First, the so-called “omitted-variable problem” often occurs in ecological studies. This issue can arise when a statistical analysis does not account for all possible explanatory variables. Second, as with previous studies’ limitations, the accuracy of the data may be questioned. Although the data were derived from established organizations and databases, errors may have occurred in data entry to introduce potential bias in our study. There were no studies to evaluate the systemic error of the government database, thus study interpretation should be done with care. Third, there exists the potential for ecological fallacy [45,46]. This property is based on the assumption that study groups are homogenous; however, in reality, individual levels are different from the ecological level, and our data should not be evaluated on an individual level. Further, an ecological approach may not be sufficient, and multi-level approaches, such as individual- and community-level studies, would be necessary to strengthen and explain the findings in this study. Fourth, socio-demographic data for the general population were analysed as a whole, including older road users, because the data for older road users could not be separated from the general population. So, in this study, we could not determine specific socio-demographic factors for traffic injury among older road users.

## Conclusions

In conclusion, the effects of socio-demographic factors on traffic injury and traffic mortality in Japan were similar for both the total population and older road users. Income per capita was preventive for traffic mortality for the total population and for older road users, while alcohol consumption was negatively associated with traffic injury and traffic mortality. The study results suggest that injury preventive measures designed for the general population will also be beneficial for older road users in Japan.

## Competing interests

The authors declare that they have no competing interests.

## Authors' contributions

TN, HB, and MH originated and designed the study. AT conducted the data analysis. TN, AT, HB, and MH interpreted the results and commented on the draft of the article. All authors approved the final version and accept responsibility for the paper.

## Research funding

Financial incentives were provided to the corresponding author from Japan Medical Association Research Institute.

## Pre-publication history

The pre-publication history for this paper can be accessed here:

http://www.biomedcentral.com/1471-2458/12/887/prepub
